# COVID-19 and *Pneumocystis jirovecii* Pulmonary Coinfection—The First Case Confirmed through Autopsy

**DOI:** 10.3390/medicina57040302

**Published:** 2021-03-24

**Authors:** Ionuț Isaia Jeican, Patricia Inișca, Dan Gheban, Flaviu Tăbăran, Maria Aluaș, Veronica Trombitas, Victor Cristea, Carmen Crivii, Lia Monica Junie, Silviu Albu

**Affiliations:** 1 Department of Anatomy and Embryology, Iuliu Hatieganu University of Medicine and Pharmacy, 400006 Cluj-Napoca, Romania; jeican.ionut@umfcluj.ro (I.I.J.); bianca.crivii@umfcluj.ro (C.C.); 2 Department of Head and Neck Surgery and Otorhinolaryngology, University Clinical Hospital of Railway Company, Iuliu Hatieganu University of Medicine and Pharmacy, 400015 Cluj-Napoca, Romania; veronicatrombitas@gmail.com (V.T.); silviualbu63@gmail.com (S.A.); 3 Department of Pathology, County Emergency Hospital Deva, 330084 Deva, Romania; patricia.bilei@gmail.com; 4 Department of Pathology, Iuliu Hatieganu University of Medicine and Pharmacy, 400006 Cluj-Napoca, Romania; dan.gheban@umfcluj.ro; 5 Department of Pathology, University of Agricultural Sciences and Veterinary Medicine, 400372 Cluj-Napoca, Romania; alexandru.tabaran@usamvcluj.ro; 6 Department of Oral Health, Iuliu Hatieganu University of Medicine and Pharmacy, 400012 Cluj-Napoca, Romania; 7 Department of Immunology, Iuliu Hatieganu University of Medicine and Pharmacy, 400162 Cluj-Napoca, Romania; victor.cristea@umfcluj.ro; 8 Department of Microbiology, Iuliu Hatieganu University of Medicine and Pharmacy, 400349 Cluj-Napoca, Romania; mjunie@umfcluj.ro

**Keywords:** COVID-19, SARS-CoV-2, *Pneumocystis jirovecii*, pneumonia, coinfection, autopsy

## Abstract

*Background*: Establishing the diagnosis of COVID-19 and *Pneumocystisjirovecii* pulmonary coinfection is difficult due to clinical and radiological similarities that exist between the two disorders. For the moment, fungal coinfections are underestimated in COVID-19 patients. *Case presentation*: We report the case of a 52-year-old male patient, who presented to the emergency department for severe dyspnea and died 17 h later. The RT-PCR test performed at his admission was negative for SARS-CoV-2. Retesting of lung fragments collected during autopsy revealed a positive result for SARS-CoV-2. Histopathological examination showed preexisting lesions, due to comorbidities, as well as recent lesions: massive lung thromboses, alveolar exudate rich in foam cells, suprapleural and intra-alveolar *Pneumocystis* *jirovecii* cystic forms, and bilateral adrenal hemorrhage. *Conclusion*: COVID-19 and *P.* *jirovecii* coinfection should be considered, particularly in critically ill patients, and we recommend the systematic search for *P. jirovecii* in respiratory samples.

## 1. Introduction

Severe acute respiratory syndrome coronavirus-2 (SARS-CoV-2), commonly referred to as coronavirus disease 2019 (COVID-19), has become a worldwide public health emergency. Although SARS-CoV-2 has caused millions of infections and hundreds of thousands of deaths in the world, pathophysiological mechanisms of the disease are not yet elucidated. The analysis of clinical cases information is useful, helping to improve medical practice and to prevent complications and coinfections [1].

Approximately 5% of patients with COVID-19 and 20% of inpatients develop acute respiratory distress syndrome (ARDS), septic shock and/or multiple organ failure, requiring intensive care treatments [2]. The mortality of patients admitted to the intensive care units reaches 40% [3]. The knowledge of coinfections associated with COVID-19 is relevant in reducing morbidity and mortality [4,5].

*Pneumocystis jirovecii* (previously called *Pneumocystis carinii* f. sp. hominis) is an atypical unicellular fungus and an opportunistic pathogen [6]. In immunosuppressed patients (due to HIV, malignancies, immunosuppressive therapy, organ transplantation, or congenital immunodeficiencies), *Pneumocystis* can cause severe pneumonia—known as *Pneumocystis* pneumonia or pneumocystosis [7]. *Pneumocystis* pneumonia is rarely found in patients with active and competent immune systems: they can be infected with *Pneumocystis*, but usually they are asymptomatic or the disease manifests as a mild respiratory infection. The first infection occurs usually in early life [8].

The occurrence of COVID-19 and *Pneumocystis* coinfection cases has previously been reported based on laboratory diagnosis [4,9,10,11] and seems to be underestimated in clinical practice [11].

Clinically, COVID-19 infection and *Pneumocystis* pneumonia can share numerous common features [10,12,13]. Computed tomography (CT) of the chest shows bilateral symmetrical ground-glass opacities in both [12,14]. Certainty diagnosis requires identification of *Pneumocystis* in lung tissue, or in lower airway fluids [13].

The diagnosis of COVID-19 and *Pneumocystis* coinfections is challenging due to overlapping clinical and radiological features, as well as limitations in laboratory diagnostics.

In this paper, we report a case of COVID-19 and *Pneumocystis* coinfection confirmed through autopsy, with massive thrombosis in the pulmonary circulation and bilateral adrenal hemorrhage, deceased 17 h after presentation to the emergency department. The main conclusion is that the COVID-19 and *Pneumocystis* coinfections should be investigated, systematically, in all severe cases of COVID (patients admitted in the intensive care units), even in those without risk factors for immunosuppression.

## 2. Case Presentation

A 52-year-old male patient, a chronic smoker and drinker, a welder (exposed to occupational respiratory hazards for about 20 years), presented to the emergency department for severe dyspnea with sudden onset on the previous day, cough and right posterior chest pain, chills, and high fever (39 °C). The patient had a series of comorbidities: hepatic steatosis, chronic alcohol liver disease, hypertensive and ischemic heart disease, and essential hypertension.

Objective examination showed an agitated, tachypneic patient, with congested facies, dry skin and mucosae, harsh vesicular murmur, right basal crepitant rales, with peripheral oxygen saturation 92%, blood pressure 200/160 mmHg, ventricular rate 106 bpm, afebrile at the time of examination.

**Figure 1:**
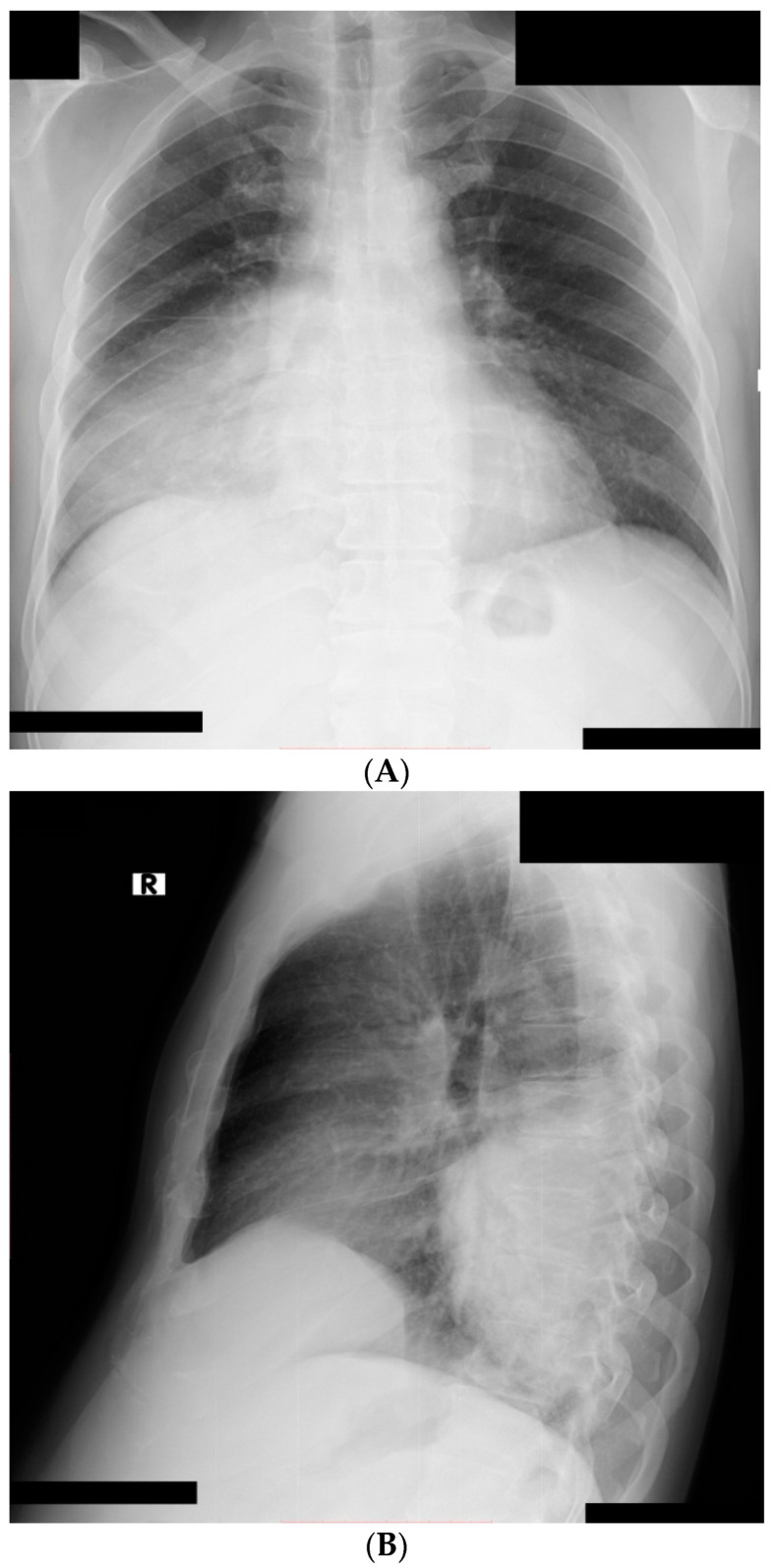
Chest X-ray: right pulmonary condensation syndrome ((**A**) postero-anterior view; (**B**) latero-lateral view).

Chest X-ray evidenced a pulmonary condensation area in the posterior inferior third of the right lung (Figure 1A,B).

Laboratory tests revealed leukopenia with lymphopenia (white blood cells 1110/μL, lymphocytes 190/μL), thrombocytopenia (127.000/μL), prolonged prothrombin time (PT) (15.6 s), international normalized ratio (INR) 1.15, hepatocytolysis (aspartate aminotransferase 88.8 units/L, alanine aminotransferase 82.8 units/L), total bilirubin 2.88 mg/dL, reduced creatinine clearance (63.8 mL/min), nitrogen retention (creatinine 1.82 mg/dL), inflammatory syndrome (C-reactive protein 450 mg/L), antibodies against HIV-1 and HIV-2 were not detectable, nonreactive anti-SARS CoV-2 antibodies. Combined throat/nasal sampling (with collection device produced by Sanimed International Impex, Bucharest, Romania) was performed for real-time PCR (RT-PCR) SARS CoV-2 (QuantStudio v5 analysis method with TaqPath COVID-19 CE-IVD RT-PCR Kit, The QuantStudioTM Design & Analysis Software, Thermo Fisher Scientific, Pleasanton, CA, USA). The result was negative. Respiratory secretions were not collected for microbiological diagnosis.

The diagnosis of right basal pneumonia, acute respiratory failure, acute chronic liver dysfunction, and acute kidney injury was established. The patient was transferred to the intensive care unit. Anti-inflammatory steroid, antibiotic, antihypertensive, oxygen therapy was initiated. The patient died 17 h after presentation.

The necropsy was performed 12 h after death. Necropsy exam revealed the following: about 1700 mL of serous fluid in the right pleural cavity, with gray deposits, which were also present focally on the surface of the right pleura, and about 500 mL of serous citrine fluid in the left pleural cavity. The middle lower lobe of the right lung had a well-demarcated red-gray area, with small violaceous areas of increased consistency, having a dry, granular cross section surface. The adrenal glands showed bilateral medullary hemorrhage. No signs of autolysis were identified.

Given the persistence of the suspicion of SARS-CoV-2 infection, five lung fragments (0.5/0.3 cm) were collected by cutting with scalpel from the red-gray area, middle lower lobe (with collection device produced by Sanimed International Impex, Bucharest, Romania). RT-PCR testing was performed on these lung fragments and the result was positive. Total RNA isolation was performed with NucleoSpin RNA for tissue (Macherey-Nagel, Dueren, Germany) according to the manufacturer’s instructions. The E gene assay (Envelope gene) was used as the first line screening tool (Ct 23) then followed by confirmatory testing with an RdRp gene assay (Ct 25.3) (RNA-dependent RNA polymerase gene—inside the Orf1ab polyprotein gene) and N gene assay (Ct 26.5) according to the Charite/Berlin protocol [15].

For the histopathological examination, we used an Olympus BX40 microscope (Tokyo, Japan) with an Olympus Camedia 4040 photo camera, hematoxylin-eosin (HE) stain (staining kit produced by Laboratorium, Bucharest, Romania), Giemsa stain (staining kit produced by Merck, Darmstadt, Germany), periodic acid–Schiff (PAS) stain (staining kit produced by Merck, Darmstadt, Germany), and Grocott stain (staining kit produced in our own laboratory). For immunohistochemistry, CD3 (Dako, polyclonal rabbit anti-human) and CD45 (Dako, monoclonal mouse antihuman, clone 2B11 + PD7/26) were used. The immunolabeling reaction was carried out by an automated immunostainer (Leica Bond-Max, Melbourne, Victoria, Australia) using a polymer-based detection system (Leica Biosystems, Melbourne, Victoria, Australia) with 3,3′-diaminobenzidin (DAB) as the chromogen.

The histopathological examination of the case evidenced preexisting lesions in various organs, which could be assigned to preexisting morbidities, favoring the severe evolution of COVID-19 infection. The histopathological examination also detected recent lesions occurring in the context of COVID-19 infection.

**Figure 2:**
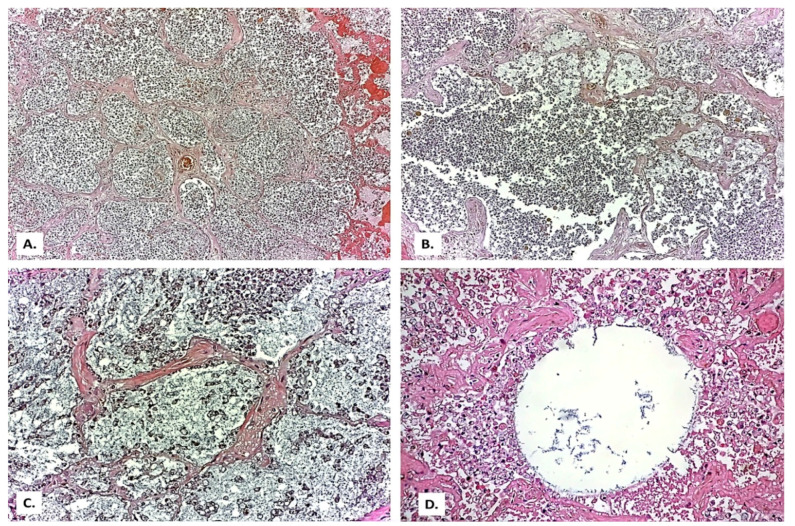
(**A**) General appearance of diffusely thickened alveolar septa (HE × 40). (**B**) Emphysema bubble surrounded by thick fibrous septa (HE × 100). (**C**,**D**) Thickened septa with myofibroblasts (HE × 200). In all images, the presence of abundant intra alveolar inflammatory exudate can be observed.

In the lung tissue, a background of lung fibrosis (Figure 2A), with emphysematous areas (Figure 2B) and thickened septa with myofibroblasts (Figure 2C,D) was seen.

**Figure 3:**
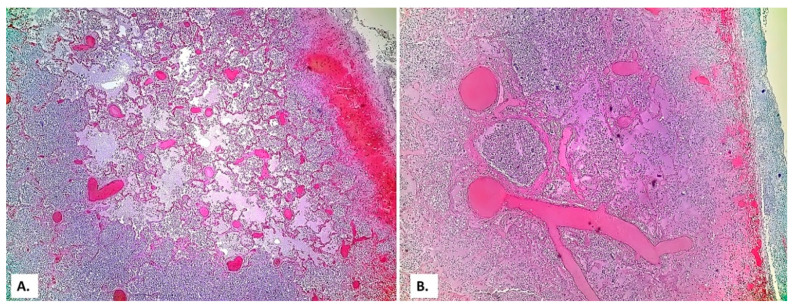
(**A**) Extensive vascular thromboses in the venous territory and a pulmonary edema focus below a subpleural hemorrhage area. (**B**) Peribronchial pulmonary artery thromboses (a bronchial lumen is seen in the center of the image). Severe stasis with subpleural hemorrhage and the presence of basophilic exudate on the surface of the pleura (HE × 40).

**Figure 4:**
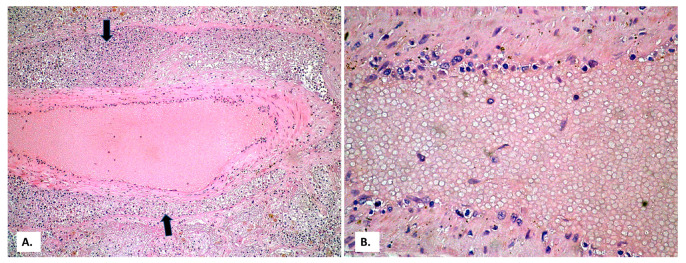
(**A**) Thrombosed arterial vessel, dilated perivascular lymphatics (arrows) filled with a cell-rich lymph fluid (HE × 40). (**B**) The same vessel, at a high magnification, allows evidencing rare lymphocytes among the endothelial cells (HE × 400).

On this fibrosis background, the large and small arterial lung vessels show many thromboses (Figure 3). The thromboses are accompanied by subpleural hemorrhagic foci (Tardieu spots, resulting from acute hypoxia), as well as by acute pulmonary edema foci in the rare alveoli unoccupied by inflammatory exudate. In the thrombosed vessels, lymphocytes adherent to the endothelium, with a significance of endotheliitis (Figure 4) but with low intensity, can be seen. In the dilated perivascular lymphatics, there is a cell-rich lymph fluid, with a cellularity similar to that of alveolar exudate (Figure 4A).

**Figure 5:**
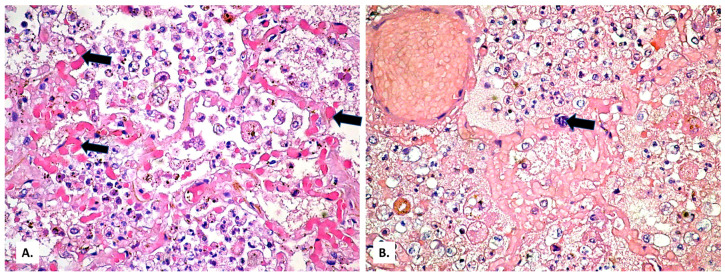
(**A**) Hyaline microthrombi (formed by fibrin) in the alveolar capillaries (arrows). (**B**) Basophilic microthrombus (formed by platelets) in the alveolar capillaries (arrows) (HE × 200).

Eosinophilic (Figure 5A) and basophilic microthrombi (Figure 5B) can also be observed, which represent aspects of disseminated intravascular coagulation (DIC).

**Figure 6:**
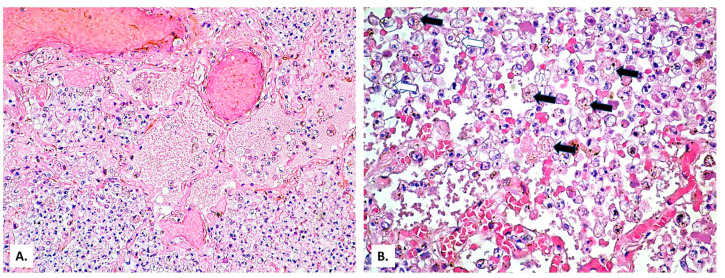
(**A**) Zonally different aspects of alveolar exudate (HE × 100). (**B**) Alveolar exudate rich in foamy macrophages (black arrows) and cystic forms (white arrows) (HE × 400).

**Figure 7:**
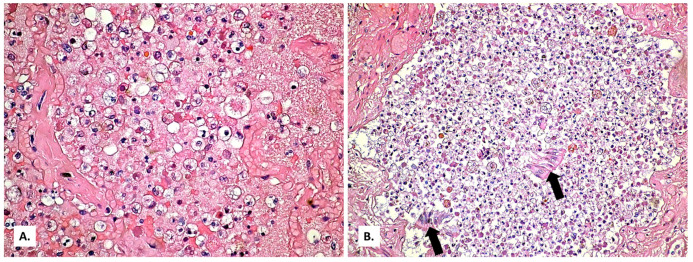
(**A**) Alveolus presenting a thickened wall with fibroblasts and abundant foamy macrophage content, on a background of granular eosinophilic material that fills the alveolus (HE × 400). (**B**) The exudate abundant in foamy macrophages also reaches the bronchial lumen which shows desquamated epithelium (arrows) (HE × 100).

**Figure 8:**
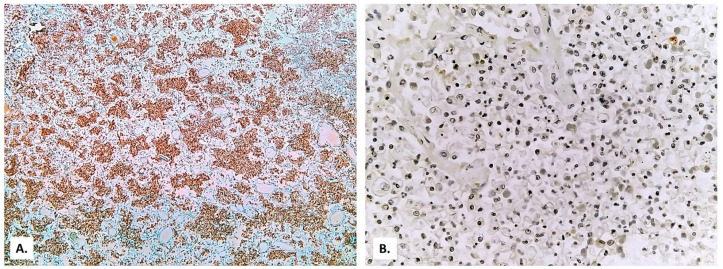
(**A**) Immunolabeling with leukocyte common antigen (CD45) (×40). (**B**) Immunolabeling with CD3 (×200).

**Figure 9:**
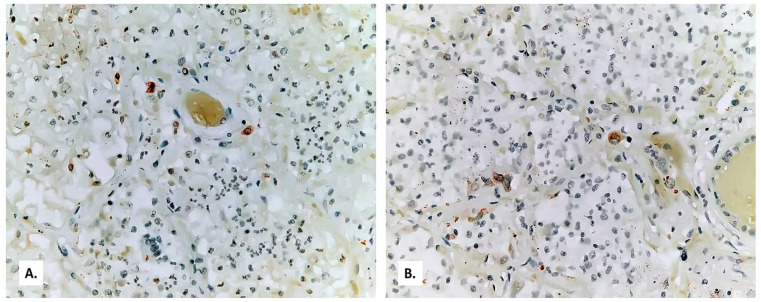
Immunolabeling for iNOS positive only in some macrophages ((**A**) microscopic field 1; (**B**) microscopic field 2).

The alveolar exudate is rich in foam cells (Figure 6 and Figure 7). Alveolar exudate immunolabeling is positive for the leukocyte common antigen (CD45, a commonly used marker of hematopoietic cells except for erythrocytes and platelets) (Figure 8A), but negative for CD3 (a common antibody for identifying T cells) (Figure 8B). Immunolabeling of the innate immune system cells for induced nitric oxide (iNOS) is extremely weak (Figure 9).

**Figure 10:**
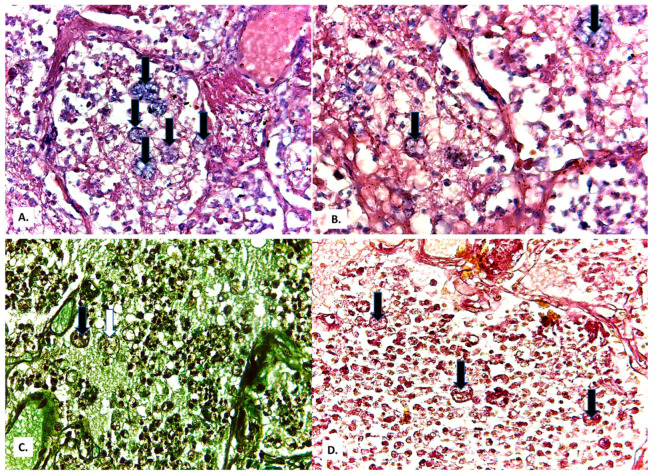
(**A**,**B**) Cystic forms positive by Giemsa stain, (**C**) Grocott-Gomori’s methenamine silver stain, and (**D**) PAS stain (black arrows—full cystic forms; white arrow—empty cystic form).

In the alveoli filled with macrophages, cystic forms with multinuclear content were also present (Figure 6B). These proved to be positive by Giemsa stain (Figure 10A,B), Grocott-Gomori’s methenamine silver stain (Figure 10C), and PAS stain (Figure 10D).

**Figure 11:**
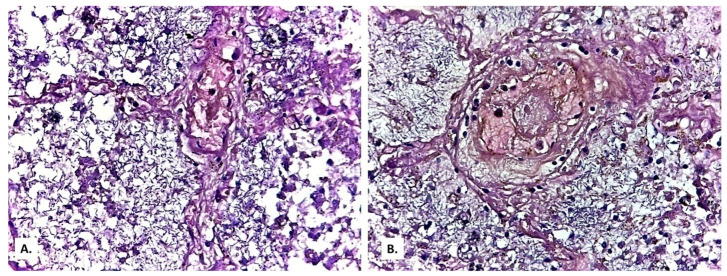
Filamentous bacterial invasion (Giemsa × 400) ((**A**) microscopic field 1; (**B**) microscopic field 2).

Giemsa stain also evidenced the fact that focally, some alveoli were invaded by a population of filamentous bacteria, in those areas the alveolar exudate having a bronchopneumonic, bacterial superinfection appearance (Figure 11).

**Figure 12:**
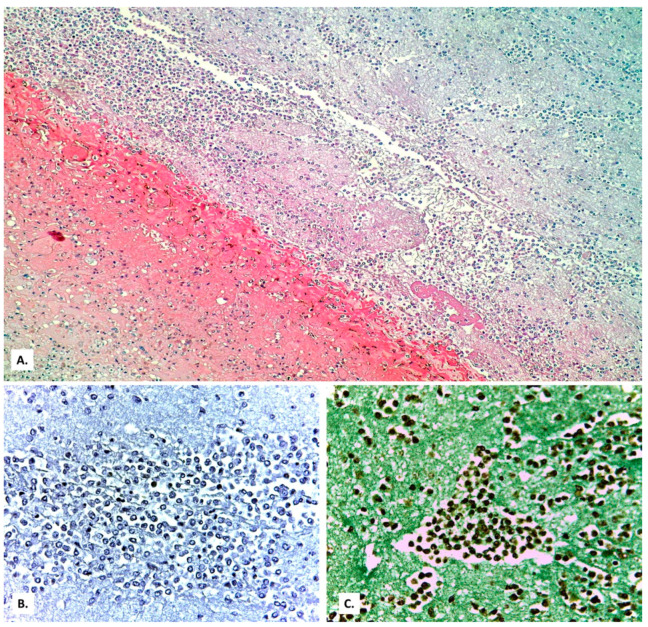
(**A**) Eosinophilic filaments of fibrin at the contact with the pleura, covered by a cell-rich basophilic material (HE × 100). (**B**) Giemsa stain and (**C**) Grocott-Gomori’s methenamine silver stain are positive.

In the pleura, the whitish exudate described macroscopically was a mixture of fibrin and basophilic granular material, rich in round cells with vesicular nuclei. These cells were positive by Giemsa and Grocott staining, supporting the diagnosis of pneumocystosis (Figure 12). Starting from these pleural images suggestive of *Pneumocystis*, it was inferred that the foamy alveolar material phagocyted by the activated macrophages was probably identical to this basophilic granular material, and the cystic forms described in Figure 10 were *Pneumocystis* cystic forms.

**Figure 13:**
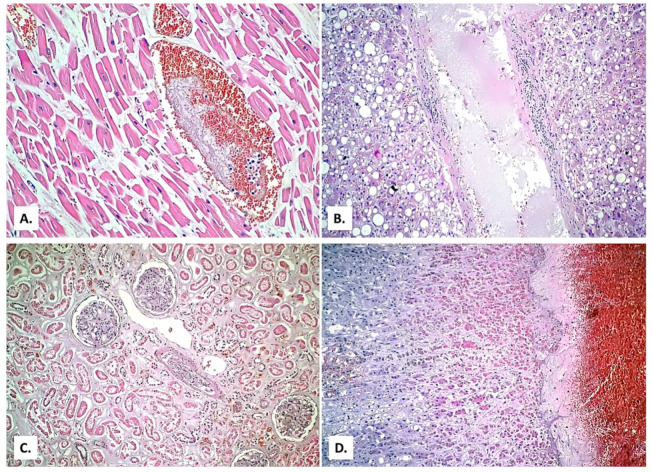
(**A**) Hypertrophic and fragmented myocardial fibers with a microthrombus in a venous vessel (HE × 400). (**B**) Liver with severe alcoholic steatosis and inflammation of the suprahepatic vein wall (HE × 100). (**C**) Kidney with tubular necrosis (HE × 200). (**D**) Adrenal medullary hemorrhage (HE × 40).

The myocardial muscle fibers are hypertrophied and fragmented, and in the medium intramyocardial venous vessels, rare microthrombi are present (Figure 13A). In the liver, alcoholic steatosis is seen. In the medium and large branches of the suprahepatic veins, there is inflammatory infiltrate which allows the diagnosis of phlebitis (Figure 13B). The kidney presents diffuse tubular necrosis (shock kidney) (Figure 13C), and in the adrenal glands, massive medullary hemorrhage can be observed (Figure 13D). The kidneys (mainly located within the interstitium) and liver (portal spaces and sinusoids) are CD45 positive.

## 3. Discussions

Respiratory viral infections predispose to the development of secondary bacterial and fungal infections [16]. Approximately 30% of COVID-19 patients could develop secondary pneumonia without an identified etiology [17].

Immunological imbalances produced in COVID-19 increase the risk of serious fungal infections (invasive pulmonary aspergillosis, invasive candidiasis or *Pneumocystis* pneumonia). Reported cases of invasive fungal coinfections were rare, probably due to the small number of bronchoscopies and necropsies performed so far [18]. Therefore, for the moment, fungal coinfections are underestimated. A multicenter cohort study of invasive fungal infections in intensive care COVID-19 patients show an incidence of 26.7% [19].

**Pathogenesis and immune response**. In the life cycle of *Pneumocystis* there are two morphological forms: the cystic form, or ascus, and the trophic form (formerly called trophozoite—from Greek, “animal that feeds”)—pleomorphic [6].

Cystic form (Figure 10) is the infective form, with a thick protective cell wall, rich in β-glucans, enabling the parasite to survive in the outside environment—the dormant stage. After respiratory inhalation, the cystic form is deposited in the alveoli and release spores, from which trophic forms develop—the active stage, in the host, associated with pathogenesis [6,20].

*Pneumocystis* is a pathogen commonly found in patients with defects in T cell immunity, especially CD4+ lymphopenia [12,21,22]. In patients with severe SARS-CoV-2 infection, lymphopenia is a common feature, with drastically reduced numbers of CD4+ [2].

In the most part of individuals, *Pneumocystis* is dormant and sparsely dispersed in the lung, without an apparent response from the host (latent infection) [13]. Considering Menon [9], we believe that SARS-CoV-2 infection led to a state of functional immune suppression related to CD4+ lymphopenia, which predisposes to the activation and proliferation of the *Pneumocystis*, with the breakout of *Pneumocystis* pneumonia. Also, COVID-19 patients could develop acute respiratory distress syndrome (ARDS), requiring steroids and immunomodulatory therapies, well-known susceptibility factors for developing *Pneumocystis* pneumonia. In our patient, no risk factors for *Pneumocystis* pneumonia were identified.

Patients with severe COVID-19 pneumonia also present lower CD3, monocyte, CD8, and CD45 counts. CD45 is necessary for T cell activation [1]. In this case, alveolar exudate immunolabeling was positive for CD45, but negative for CD3. This fact indicates a severe lack of immune response from T lymphocytes, which results in a functional deficit translating into very weak immunolabeling of the innate immune system cells—macrophages, neutrophils, and natural killer cells—for iNOS [23], see Figure 9.

The functional deficit due to the absence of T lymphocytes translates into a compensatory activation of the function of macrophages (type 2 pneumocytes and blood macrophages). Macrophages become foamy (Figure 6B) due to the fact they have intensely phagocyted a preexisting foamy alveolar material, releasing in this process a wide variety of proinflammatory cytokines (cytokine storm) [23]. Again, *Pneumocystis* elicits a response of the alveolar macrophages and phagocytosis [13]. Thus, both pathogens cause a massive activation of macrophages, which can be observed in our case as well: most of the alveoli have a cell content with rare lymphocytes, but rich in foamy macrophages. Foamy macrophages were identified in COVID-19 infection also by other authors [24].

Due to the lack of identification of another prothrombogenic factor, in this case, we consider COVID-19 infection as a determinant of the breakout of massive pulmonary thrombosis. Coagulopathy associated with COVID-19 is a combination of low grade DIC and localized pulmonary thrombotic microangiopathy [25].

In our patient, pulmonary fibrosis and emphysematous areas were interpreted to be lesions preceding SARS-CoV-2 infection. In addition, the patient, a welder, has long been exposed to occupational respiratory hazards. Respiratory damage may have contributed, over time, to the development of pulmonary fibrosis on a toxic basis. However, three other COVID-19 positive patients, with various comorbidities, who died in the same hospital and were examined, presented pulmonary fibrosis with different degrees of severity, and in one of them we could notice the presence of Hamman-Rich syndrome (also known as acute interstitial pneumonia [26]), in which pulmonary fibrosis was developed very rapidly, on the background of COVID-19 infection.

COVID-19 infection is associated with bilateral adrenal hemorrhage [27,28], which can also be identified in our case (Figure 13D).

**Clinical Picture**. Clinically, COVID-19 infection and *Pneumocystis* pneumonia have numerous common features: fever, fatigue, dry cough, tachypnea, dyspnea, desaturation, and relatively normal chest auscultation [10,12,13]. Evolution progresses to death in almost all cases unless treatment is administered [13].

**Imaging and Laboratory**. Multifocal ground-glass opacities are observed both in pneumonia with *Pneumocystis* and SARS-CoV-2 infection, making radiographic differential diagnosis difficult [29,30,31,32]. In our case, the radiological lung appearance showed a relatively well-demarcated lobar infiltration.

Sensitivity of RT-PCR from throat swabs to detect SARS-CoV-2 RNA has been reported to be up to 95% [15]. However, the sensitivity of RT-PCR depends on the type of specimen and the assay used [33]. It is unclear why in our patient the SARS-CoV-2 PCR testing results were negative.

Standard laboratory diagnosis involves the histological detection of *Pneumocystis* in respiratory specimens (bronchoalveolar lavage fluid, induced sputum, or lung biopsy samples), through chemical staining (Giemsa, crystal violet, and Diff-Quick stains detect both the cystic and trophic forms; toluidine blue or Grocott-Gomori’s methenamine silver stains color the walls [34]) or immunofluorescence staining with anti-*Pneumocystis* monoclonal antibodies [35].

The diagnosis of *Pneumocystis* pneumonia can be made by molecular PCR tests [36]. The significance of detected *Pneumocystis* DNA using PCR alone remains uncertain, and can be due to colonization of the respiratory tract [37]. Real-time quantitative (RTqPCR) assays are the only PCR method recommended for diagnosis [38]. A study conducted in 108 critical patients with COVID-19 from the intensive care unit showed that 9.3% had positive *Pneumocystis* RTqPCR tests from bronchoalveolar lavage/tracheal aspirate/sputum samples [11].

Serological tests for detection of anti-*Pneumocystis* antibodies have not proven clinically useful for establishing a diagnosis of pneumonia [35,39]. β-1,3-glucan is sensitive, but not specific [38,40]. The identification of specific biomarkers would be particularly useful, especially in patients where manipulation of the airway is limited and therefore the collection of respiratory samples is difficult.

In this patient, the diagnosis of *Pneumocystis* pneumonia was established through histological detection in lung fragments. The lack of confirmation of the presence of *Pneumocystis* in lung fragments and by molecular diagnosis PCR is a limitation of our study. However, the identification of *Pneumocystis* cystic forms in four distinct histological stains, from four different paraffin blocks, greatly reduces the possibility that they were artifacts.

**Clinical Management**. The firstline drug for the treatment of *Pneumocystis* pneumonia is trimethoprim-sulfamethoxazole administered orally or intravenously. For patients who cannot tolerate this treatment, or if it is inefficient, the alternative is clindamycin-primaquine, dapsone-trimethoprim, intravenous pentamidine, and atovaquone. Prophylaxis with trimethoprim-sulfamethoxazole, or cotrimoxazole, dapsone, atovaquone, and aerosol pentamidine is effective in preventing *Pneumocystis* pneumonia in patients at risk [41,42,43].

For COVID-19 and *Pneumocystis* coinfection, high-dose corticosteroid therapy is controversial [10] but can be a therapeutic necessity. For COVID-19 patients with *Pneumocystis* positive respiratory tests who require steroids, prophylaxis was administered to avoid the risk of pneumocystosis [11].

Table 1 presents a comparison of COVID-19 and *Pneumocystis* coinfection cases reported so far.

## 4. Conclusions

This paper is in line with those raising a red flag about COVID-19 and *Pneumocystis* coinfection, which should be seriously taken into consideration, particularly, in critically ill patients, even if patients do not have classical risk factors for *Pneumocystis* pneumonia. Hence, we recommend a systematic search for *Pneumocystis* in respiratory samples for COVID-19 critically ill patients. Tests combining molecular techniques and biomarkers could optimize the diagnosis of *Pneumocystis* pneumonia in patients with diagnostic suspicion.

## Figures and Tables

**Table 1:** Reported COVID-19 and *Pneumocystis* coinfection cases.

	Clinical Picture	Imaging Diagnosis	Laboratory Diagnosis	Treatment	Clinical Evolution
Male patient, 25-year-old [4]. Comorbidities: HIV (new diagnosis)	Profound hypoxemia	Chest X-ray: large right pneumothorax and extensive interstitial disease. Chest CT: apical cystic changes, diffuse ground-glass opacities, dense consolidation, and pneumothorax.	Nasopharyngeal SARS-CoV-2 PCR: positive. HIV serology: positive. *Pneumocystis* antigen (bronchial aspirate): positive.	Intubated. Trimethoprim-sulfamethoxazole, prednisone, and remdesivir.	Improved clinically, extubated 21 days later.
Female patient, 83-year-old [9]. Comorbidities: asthma, valvulopathy, ulcerative colitis.	Fever (39.3 °C), malaise, headache, dry cough, and dyspnea (SpO_2_ 86%)	Chest CT: diffuse bilateral ground-glass opacities and small nodular foci of consolidation.	Leukocytosis, lymphocytopenia. Nasopharyngeal SARS-CoV-2 PCR: positive. HIV serology: nonreactive. *Pneumocystis* PCR (tracheal aspirate): positive. β-d-glucan elevated (305 pg/mL).	Intubated. Trimethoprim-sulfamethoxazole.	Improved clinically, extubated 7 days later.
Male patient, 55-year-old [10]. Comorbidities: controlled HIV and asthma.	Fever, cough, and hypoxia	Chest CT: Extensive subpleural and para-mediastinal cystic changes, subpleural ground-glass changes bilaterally.	Nose and throat SARS-CoV-2 PCR (day 2): negative. Throat SARS-CoV-2 PCR (day 7): positive. *Pneumocystis* PCR (sputum): positive.	High-flow oxygen, no intubation. Cotrimoxazole, prednisolone.	Improved clinically, discharged on day 14.
Male patient, 36-year-old [44]	Shortness of breath, fever (38.7 °C), nausea, and diarrhea for 3 weeks	Chest X-ray: diffuse hazy pulmonary opacifications. Chest CT: ground-glass alveolar airspace disease.	Lymphocytopenia Oropharyngeal SARS-CoV-2 PCR: positive. *Pneumocystis* PCR (bronchial alveolar lavage): positive. β-d-glucan elevated (>500 pg/mL).	Intubated, started with remdesivir and antibiotics. Trimethoprim-sulfamethoxazole, prednisone, remdesivir, and COVID-19 convalescent plasma.	Cardiac arrest 26 days later. No autopsy.

## Data Availability

The study did not report any data, apart from those reported already in the article.
